# In Silico Analysis and Biochemical Characterization of *Streptomyces* PET Hydrolase with Bis(2-Hydroxyethyl) Terephthalate Biodegradation Activity

**DOI:** 10.4014/jmb.2404.04030

**Published:** 2024-07-25

**Authors:** Gobinda Thapa, So-Ra Han, Prakash Paudel, Min-Su Kim, Young-Soo Hong, Tae-Jin Oh

**Affiliations:** 1 Department of Life Science and Biochemical Engineering, Sun Moon University, Asan 31460, Republic of Korea; 2 Bio Big Data-Based Chungnam Smart Clean Research Leader Training Program, Sun Moon University, Asan 31460, Republic of Korea; 3 Chemical Biology Research Center, Korea Research Institute of Bioscience and Biotechnology, Ochang 28116, Republic of Korea; 4 Genome-Based BioIT Convergence Institute, Asan 31460, Republic of Korea; 5 Department of Pharmaceutical Engineering and Biotechnology, Sun Moon University, Asan 31460, Republic of Korea

**Keywords:** PET hydrolase, enzymatic BHET degradation, *Streptomyces*, bis(2‐hydroxyethyl) terephthalate (BHET), MD simulation

## Abstract

Polyethylene terephthalate (PET), one of the most widely used plastics in the world, causes serious environmental problems. Recently, scientists have been focused on the enzymatic degradation of PET, an environmentally friendly method that offers an attractive approach to the degradation and recycling of PET. In this work, PET hydrolase from *Streptomyces* sp. W2061 was biochemically characterized, and the biodegradation of PET was performed using the PET model substrate bis (2-hydroxyethyl terephthalate) (BHET). PET hydrolase has an isoelectric point of 5.84, and a molecular mass of about 50.31 kDa. The optimum pH and temperature were 7.0 and 40°C, respectively. LC-MS analysis of the enzymatic products showed that the PET hydrolase successfully degraded a single ester bond of BHET, leading to the formation of MHET. Furthermore, in silico characterization of the PET hydrolase protein sequence and its predicted three-dimensional structure was designed and compared with the well-characterized IsPETase from *Ideonella sakaiensis*. The structural analysis showed that the (Gly-x1-Ser-x2-Gly) serine hydrolase motif and the catalytic triad (Ser, Asp, and His) were conserved in all sequences. In addition, we integrated molecular dynamics (MD) simulations to analyze the variation in the structural stability of the PET hydrolase in the absence and presence of BHET. These simulations showed the formation of a stable complex between the PET hydrolase and BHET. To the best of our knowledge, this is the first study on *Streptomyces* sp. W2061 to investigate the BHET degradation activity of PET hydrolase, which has potential application in the biodegradation of plastics in the environment.

## Introduction

Plastics are synthetic polymers with the advantages of chemical resistance, lightweightness, and cost-effectiveness. Their ubiquitous utilization on a global scale since the 1950s underscores their pivotal role in contemporary daily existence [1]. Worldwide, plastic pollution has become a global environmental issue. Every year, over 350 million tons of plastic are produced globally for a wide range of applications [2]. Billions of tons of plastic waste in landfills, floating in trash islands in the ocean, and scattered as microplastics have led to severe global pollution [3]. Polyethylene terephthalate (PET) is a condensation polymer formed by an ester bond between ethylene glycol (EG) and terephthalic acid (TPA) [4]. Due to its transparency, flexibility, and resistance to natural degradation, PET is one of the most widely employed plastic products with various applications used in everyday life, including the production of beverage bottles and textiles [5]. However, a large amount of PET waste has accumulated in the environment and caused severe environmental problems [6].

Bis(2-hydroxyethyl) terephthalate (BHET) is a commercially available monomer that closely resembles the core structure of PET and has been used extensively in PET research [7]. In the enzymatic hydrolysis of PET, BHET and MHET were detected as intermediates, while TPA and EG were identified as end products [8, 9]. The conventional method for recycling PET is associated with high energy consumption and typically produces harmful by-products, using both physical and chemical processes [10]. The enzyme isolated from microorganisms has provided a novel and eco-friendly option for the development of biodegradation strategies [11]. Modern technology can not only alleviate the problem of plastic waste accumulation, but also reclaim the raw materials necessary for recycled plastics [12]. In recent years, it has been reported that certain microorganisms, such as *Fusarium oxysporum*, *Fusarium solani*, *Thermomyces insolens*, *Thermobifida alba*, *Bacillus subtilis*, and *Penicillium citrinum*, have shown the ability to degrade PET by using it as their primary carbon and energy source for cell growth [13-16]. The genus *Streptomyces*, which belongs to the Actinomycetes group, is well known to produce compounds and enzymes of ecological, industrial, and clinical interest [17–20]. The enzymes best known for their PET-hydrolyzing ability are serine hydrolases, which include carboxylesterases, cutinases, lipases and esterases [21]. PET hydrolase (IsPETase) from *Ideonella sakaiensis* [22] and cutinase from leaf-branch compost (LCC) [23, 24] are the most extensively studied PET-active enzymes and can therefore be considered as model PET hydrolases. All of these enzymes share a common catalytic triad (S-D-H) and utilize a similar reaction mechanism, but each subfamily exhibits unique substrate recognition properties [25]. Structural analyses have shown that PET hydrolase belongs to the α/β- hydrolase superfamily with the canonical α/β- hydrolase fold, comprising a central β-sheet flanked by α-helices [26]. In 2016, Yoshida *et al*. discovered a bacterium, *I. sakaiensies* 201-F6, capable of breaking down PET as an energy source using novel secreted enzymes, IsPETase and mono(2-hydroxyethyl) terephthalate hydrolase (MHETase) [22]. The enzymatic reaction mechanism of IsPETase has been studied more extensively than that of other PET hydrolases [27]. However, the degradation activities of PET hydrolases are still very weak due to the mobility of PET polymer chains, the specificity of their active sites, and the necessity for the relatively high temperature [24]. In the current research, we sought to identify a PET hydrolase in *Streptomyces* sp. W2061. The activity of the enzyme was confirmed by heterologous expression in *Escherichia coli*, demonstrating its ability to degrade BHET. Furthermore, in silico approaches combining homology modeling, docking, and MD simulations were used to investigate the flexibility of the catalytic site for substrate binding, and aid in understanding the BHET degradation activity.

## Materials and Methods

### Strains and Reagents

Bacterial strain *Streptomyces* sp. W2061 was obtained from the Chemical Biology Research Center (KRIBB, Republic of Korea). PET was purchased from Sigma-Aldrich (China), BHET was obtained from Sigma-Aldrich (Japan), and TPA was purchased from Tokyo Chemical Industry Co., Ltd. (Japan). DNA polymerase, restriction enzymes, and T-vector and T4 DNA ligase were purchased from Clontech Takara (Japan). All other analytical-grade chemicals used in this study were obtained from available commercial sources.

### Protein Homology Search and Phylogeny Analysis

A protein homology search was performed by using protein BLAST (http://blast.ncbi.nlm.nih.gov/Blast.cgi). A total of 16 potential PET hydrolase homologous protein sequences were identified from the RCSB PDB database (http://www.rcsb.org) [28]. ClustalW was used for protein sequence alignment and phylogeny analysis was performed by using MEGA11 [29]. The software ESPript 3 (http://espript.ibcp.fr) [30] was used for multiple sequence alignment of proteins. Expasy proteomics server (https://web.expasy.org/protparam) [31] was used to calculate the theoretical molecular mass and isoelectric point (pI) of the PET hydrolase. The percentage identity and similarity of PET hydrolase with similar enzymes were calculated by using the Emboss Needle pair alignment tool (http://www.ebi.ac.uk/Tools/psa/emboss_needle) [32].

### Protein Structure Analysis, Modeling, and Molecular Docking

In silico protein structure prediction was performed by using the online web-server SWISS-MODEL [33]. Molecular docking experiments were performed using AutoDock Vina, and the results were visualized by Pymol [34, 35] and Ligplot+ [36]. For the docking, the model substrate BHET (Zinc database ID: ZINC02040111) was used as the ligand [37]. BHET has already been used in molecular docking studies as a model substrate for PET degradation, using both *in vitro* and in silico approaches [38].

### Molecular Dynamics Simulation

The MD simulation for PET hydrolase alone and PET hydrolase–BHET complex were performed using GROMACS package [39]. Both systems were prepared using CHARMM36m force field [40] and TIP3P water [41]. The parameters of CHARMM36m force field for BHET were taken from CGenFF server [42]. Both systems emerged in a cubic box containing specific numbers of water molecules (13,794 for PET hydrolase alone) and (13,792 for PET hydrolase–BHET complex), for which the distance of protein from the edge of the box was kept at 1.0 nm. To preserve the overall neutrality, each system was supplemented with the required number of counterions (Na^+^ and Cl^–^) along with the concentration of 0.15 M NaCl [43]. Each system was then subjected to energy minimization utilizing the steepest descent method followed by equilibration for 500 ps under NVT and NPT conditions. Both equilibrated systems were then subjected to MD simulations of 100 ns each, while upholding a target pressure of 1 bar with Parinello-Rahman barostat [44], and a temperature at 310 K using a Nose-Hoover thermostat [45]. The LINCS algorithm was applied to constrain all the bonds [46], while the bonds within the water molecules were constrained by the SETTLE algorithm [47]. The long-range electrostatic and short-range van der Waals interactions were computed employing the particle mesh Ewald (PME) method, with a short-range interaction cut-off value of 1.2 nm [48].

After the completion of the 100 ns MD simulations, trajectories were analyzed using various GROMACS utilities for structural stability analyses through root-mean-square deviation (RMSD) and root-mean-square fluctuations (RMSF) using the gmx rms and gmx rmsf modules. The conformational clustering was carried out using the Daura *et al*. algorithm [49] with the gmx cluster utility of GROMACS at an RMSD cut-off value of 0.12 nm. Lastly, binding free energy was calculated using the molecular mechanics Poisson-Boltzmann surface area (MM-PBSA) method employing g_mmpbsa [50]. During the binding free energy calculation, the entropy calculation was not considered, in accordance with previous studies [51].

### Cloning and Heterologous Expression

The full length of the PET hydrolase gene from *Streptomyces* sp. W2061 (GenBank Accession No. 030719063.1) was designed by (Geno-Tech, Republic of Korea). The PET hydrolase was amplified by polymerase chain reaction (PCR) using the primers (forward): 5'‐GAATTC ATG CAG CAG CAC CTC CCC T‐3' (EcoRI restriction site underlined), and (reverse): 5'‐AAGCTT TCG GCT CTA CGC CGT GTG‐3' (HindIII restriction site underlined). The amplified PCR product (951 bp) was ligated into the T-vector and transformed into *E. coli* XL1-Blue. Subsequently, both the cloned plasmid and pET-32a (+) plasmid were double digested with EcoRI and HindIII and ligated using T4 DNA ligase. The presence of insert was verified via Sanger sequencing T7 promoter and T7 terminator primers. The resulting recombinant plasmid was then transformed into *E. coli* BL21(DE3) cells. For protein overexpression, a positively transformed colony was picked and grown in lysogeny LB broth containing 100 μg/ml ampicillin at 37°C, until the cell density at OD_600_ reached approximately 0.6~0.7. Protein expression was induced with 0.5 mM isopropyl-β-D thiogalactopyranoside (IPTG) and cells were incubated at -20°C for 24 h. The induced cell pellets (from a 200 ml culture) were collected by centrifugation for 30 min at 3,500 ×*g* at 4°C, washed twice with 50 mM Tris-HCL buffer (pH 8.0), and stored at -50°C for further use.

For purification, the induced cells were resuspended in 8 ml Tris-HCl buffer (pH 8.0) and disrupted using ultrasonic treatment for 15 min with 5 s pulses and 20 s pauses, using an ultrasonic homogenizer (Ulsso HiTech Co., Ltd., Republic of Korea). The cell extract was centrifuged at 11,000 ×*g* for 25 min at 4°C and the soluble fraction was applied to 2 ml Talon metal affinity resin (Takara), and incubated for 2 h at 4°C. The bound protein was poured into a gravity flow column, washed with 20 ml of Trish-HCl (pH 8.0) buffer, and eluted with different fractions of the imidazole (10, 100, and 200 mM) containing Trish-HCl (pH 8.0). The eluted protein was concentrated using Amicon centrifugal filters (Millipore, 30kDa). The purity of the protein was analyzed by 15%SDS-PAGE, and the protein concentration was measured by Bradford protein assay [52].

### Enzymatic Activity Assay

The purified protein (50 nM) was incubated in 500 μl Tris-HCl buffer (50 mM Tris-HCl, pH 7.0) containing 4 mM BHET for 18 h at 30°C. The reaction was terminated by heating at 80°C for 10 min, filtered through a 0.22 μm Whatman filter (Henke-Ject, Germany) and subsequently analyzed by HPLC. All samples were processed in triplicate, with the reaction mixture containing boiled protein considered a negative control.

### HPLC and LC-MS Analysis

The HPLC analysis was conducted on a Dionex UltiMate 3000 Diode Array Detector (Thermo Fisher Scientific, Japan) with a Mightysil Reverse-Phase Analytical Column (4.6 × 250 mm, 5 μm). The mobile phase (A) was TFA water (0.05%) and acetonitrile (B). The gradient elution program was set as follows: 30-65% (v/v) acetonitrile from 0 to 25 min, and 65-100% acetonitrile from 25 to 35 min, with an optimal flow rate of 1.0 ml/min. Each sample was injected with a volume of 20 μl, and the temperature of the column and detector was maintained at 30°C. The effluent was detected at 260 nm. LC-MS analysis was carried out using a liquid chromatography mass-spectrometer (UPLC, SYNAPT G2-S/ACQUITY, USA) in positive ion mode.

### Effects of pH, Temperature Metal Ions and Organic Solvents on Enzyme Activity

The impact of pH, temperature, organic solvents, and metal ions on enzyme activity was studied using the model substrate para-nitrophenol (p-NPC_4_). The total reaction volume was 2 ml, which included 120 μl of 10 mM (p-NPC_4_) substrate, 1.68 ml of 50 mM (Tris-HCl, pH 7), and 200 μl of purified PET hydrolase. After a 120-min reaction at 40°C, the production of (p-NPC_4_) was measured at 405 nm. One unit of PET hydrolase was defined as the amount of enzyme that produced 1 μmol of p-nitrophenol per minute.

Under the standard assay conditions, the stability of PET hydrolase was examined across pH levels (4.0-10.0) using the following buffers: 50 mM/L sodium acetate buffer pH (4.0-5.0), citrate phosphate buffer pH (5.0-6.0), sodium phosphate buffer (pH 6.0-8.0), Tris-HCl buffer pH (8.0-9.0), and glycine-NaOH buffer pH (9.0-10.0). Additionally, the optimal temperature for PET hydrolase activity was determined by evaluating enzyme activity at different temperatures between 20°C and 70°C.

Moreover, the influence of different metal ions and organic solvents on the PET hydrolase activity was investigated, based on various metal ions Na^+^ (NaCl), K^+^ (KCl), NH_4_^+^ (NH_4_Cl), Mg^2+^ (MgCl_2_), Ba^2+^ (BaCl_2_), Zn^2+^ (ZnCl_2_), Fe^2+^ (FeCl_2_), and Cu^2+^ (CuSO_4_) at a final concentration of 1 mM, and organic solvents (methanol, acetonitrile, ethyl acetate, Tween 20, DMSO, and EDTA) in a volume concentration of 1.0%. The enzyme activity without the addition of metal ions or organic solvents served as a control.

## Results and Discussion

### Protein Homology Search and Phylogeny Analysis

**Fig. 1:**
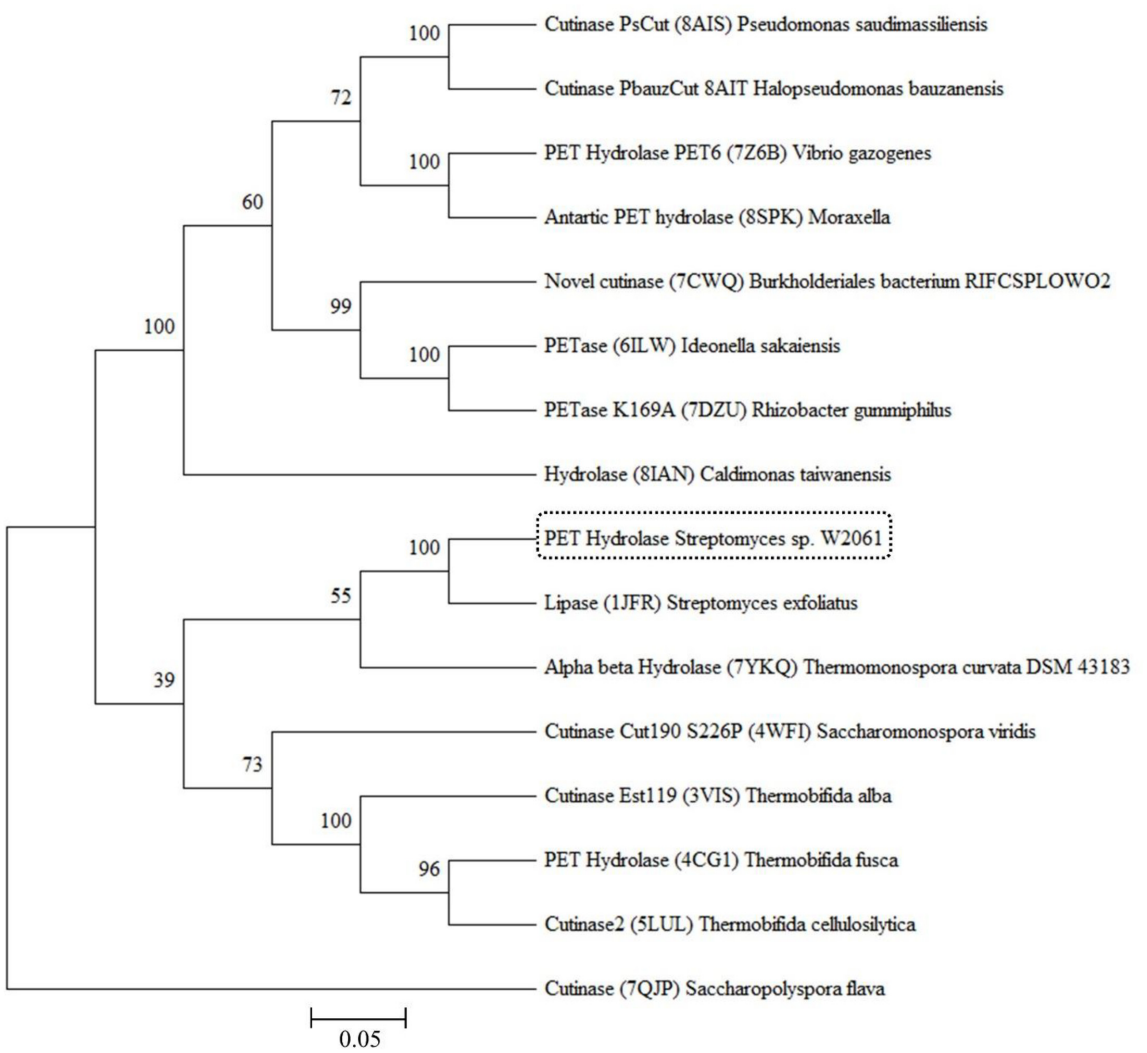
Phylogenetic tree of PET hydrolases from *Streptomyces* sp. W2061. Protein sequences were aligned using the built-in CLUSTALW (default parameters), and the tree was built using the neighbor-joining method with default parameters and 1,000 bootstrap replications.

By applying a sequence similarity search approach using the protein BLAST, the *Streptomyces* PETase (PET hydrolase)-encoding gene had a molecular weight of 50.31 kDa (GenBank accession no. WP 030719063.1)(Fig. S1), and its theoretical isoelectric point (pI) value was 5.84. A total of 16 potential PET hydrolase homologous proteins were identified from the RCSB PDB database. These proteins were used for phylogenetic analysis using MEGA11. The amino acid sequences were aligned with ClustalW [53], and a maximum likelihood phylogenetic tree was generated with 1,000 bootstrap replicates with a cut-off value of 50%, as shown in (Fig. 1). It appears that all enzymes in the tree had a common ancestral origin, except for cutinase (7QJP). There are 4 clades, and our PET hydrolase was closely related to the lipase (1JFR) of *Streptomyces exfoliates* (Fig. 1). Using InterProScan and Pfam, the PET hydrolase was identified as a member of the α/β hydrolase family. Most polyesterases belong to this superfamily, which includes enzymes that have similar structures but perform a variety of functions [54].

The amino acid sequences of the PET hydrolase were homologous to lipase (1JFR) from *Streptomyces exfoliatus*, which has 78.8% identity and 81.3% similarity. The lowest identity (48.4%) and similarity (58.4%) were found with a PET hydrolase (4CG1) from *Thermobifida fusca* (Table S1). There was 55.1% identity and 64.7% similarity with cutinase Est119 (3VIS) from *Thermobifida alba*, and 56.3% identity and 66.2% similarity with an alpha-beta-hydrolase (7YKQ) from *Thermomonospora curvata* DSM (Table S1). These differences in the degrees of similarity of these PET hydrolases indicate the presence of structural adaptation elements of the enzymes to ensure a balance between their activity, stability, and flexibility required for catalysis [55]

**Fig. 2:**
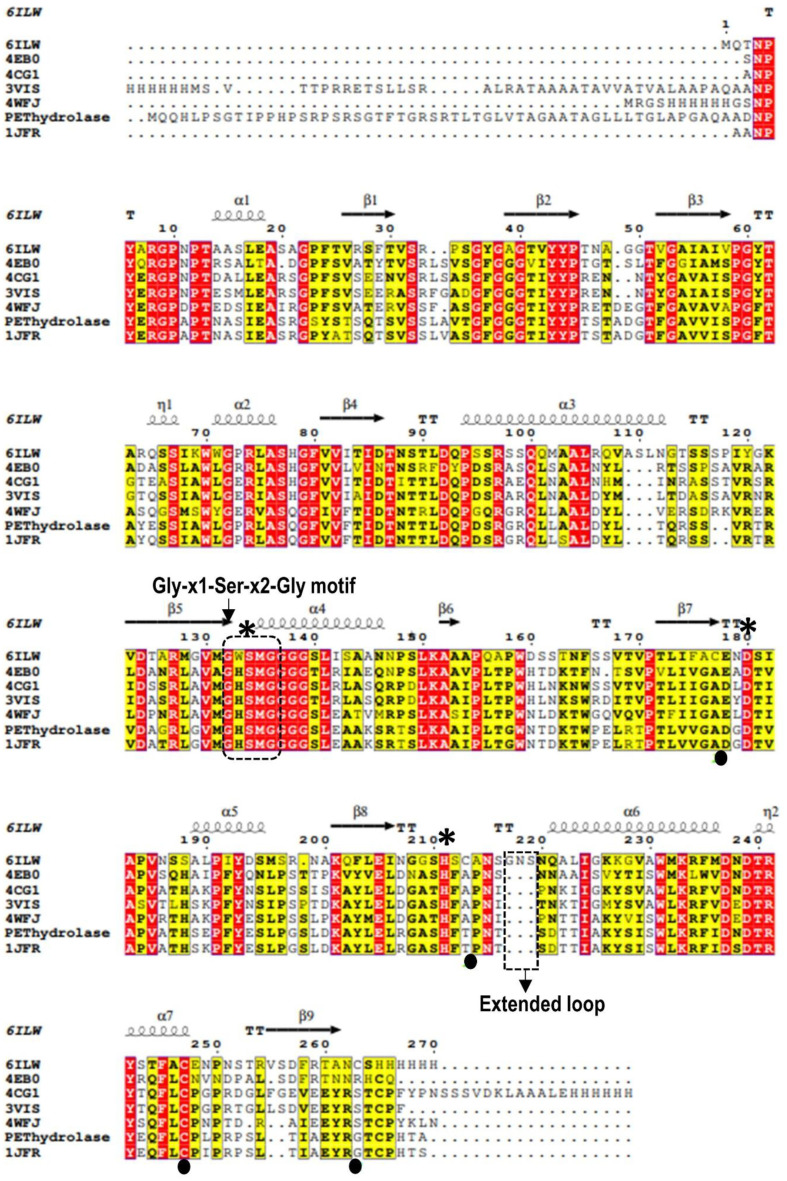
Structure-based sequence alignment of PET hydrolase with its structure-available homologues (6ILW, *Ideonella sakiensis*; 4EB0, leaf-branch compost bacterial; 4CG1, *Thermobifida fusca*; 3VIS, *Thermobifida alba*; 4WFJ, *Saccharomonospora viridis*; and 1JFR, *Streptomyces exfoliatus*). PDB codes are indicated. The catalytic triad residues are indicated by asterisk. Motif and extended loop are indicated by black dotted box. Residues mutated in PET hydrolase are shaded in yellow.

Multiple sequence alignments were employed to identify the conserved regions, motifs, and active sites of the enzyme, based on sequence comparisons with other PET hydrolases. The MSA of the PET hydrolase revealed a typical catalytic triad consisting of Ser-Asp-His, which was to be expected as this triad is known to be essential for the enzymatic activity of this enzyme class. A consensus motif (G‐X‐S‐X‐G) was identified around the serine in the active site (Fig. 2). Serine acts as a nucleophilic center for the hydrolysis of ester, amide, or thioester bonds. The histidine residue serves as a general base or acid, while the carboxylate group of aspartic acid helps to properly orient and neutralize the charge of the imidazole ring of histidine [56]. The deprotonated catalytic serine attacks the carbonyl bond of the substrate, leading to the cleavage of one of the acyl–oxygen bonds [57].

### Protein 3D Structure Prediction and Molecular Docking

**Fig. 3:**
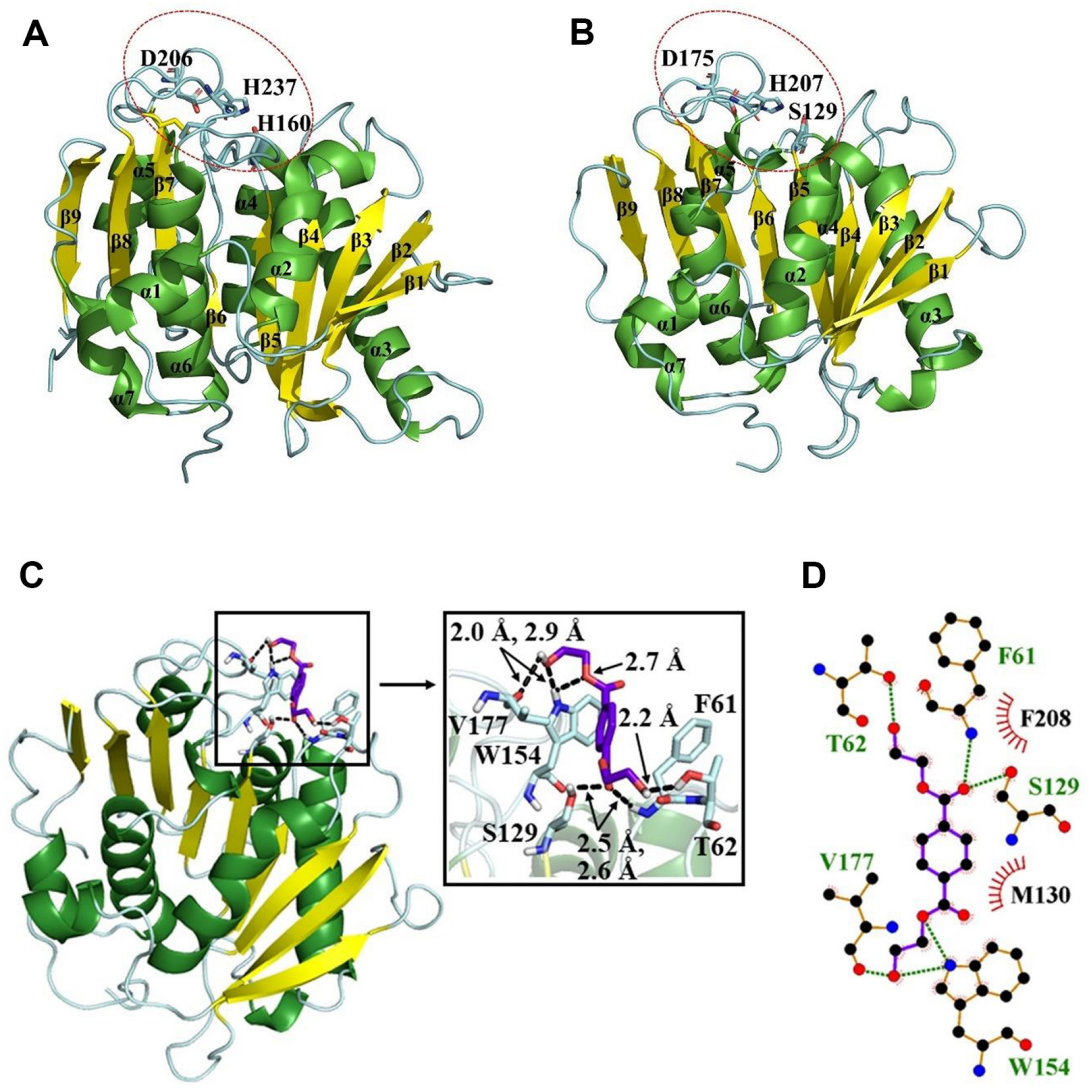
Three-dimensional protein structure comparison and molecular docking. (**A**) IsPETase three-dimensional structure (PDB ID: 6ILW), the catalytic triad (Ser160, Asp206, and His237) is circled in red. (**B**) Predicted three-dimensional structure of the PET hydrolase protein, generated using SWISS-MODEL, with the crystal structure of lipase from *Streptomyces* sp. as template (PDB ID: 1JFR). The catalytic triad (Ser129, Asp175, and His207) is circled in red. (**C**) The docked complex depicting hydrogen bond interactions of BHET with PET hydrolase. (**D**) The 2D interaction map highlighting the hydrogen bond (green dotted lines) and hydrophobic contacts (red semicircles) between the residues of PET hydrolase and BHET.

The conformation of a protein plays a crucial role in determining its function and stability [58]. To gain a deeper understanding of the possible functional characteristics and structural arrangement of the PET hydrolase protein, an in silico prediction of its three-dimensional structure was performed using SWISS-MODEL. The lipase from *Streptomyces exfoliates* (PDB ID: 1JFR) was used as a template for model prediction. The generated model has a GMQE score and QMEN Z-score of 0.78 and -0.04, respectively, which indicates a reliable predicted model (Fig. 3B). When compared to the structure of IsPETase (Fig. 3A), the predicted structure of the PET hydrolase shows many similarities. Both belong to the same α/β-hydrolase superfamily and have 9 β-sheets and 7 α-helices [59]. The configuration of the catalytic triad was also similar, as highlighted in Fig. 3A and 3B, which may partially explain the activity of these enzymes in the degradation of synthetic polyesters. Regarding the potential differences observed between these two protein structures, the most striking differences were the lack of a disulfide bond and an extended loop between α6 and β8 in our PET hydrolase [7].

To anticipate how the protein binds with the ligand, a molecular docking experiment was performed using a predicted model of the PET hydrolase and the ligand BHET with the program AutoDock Vina. The poses of the most favored conformation of BHET showed a binding energy of ≤ -5.1 kcal/mol, which was similar to those previously described in similar molecular docking experiments with IsPETase and BHET [18]. The binding modes were analyzed in detail using PyMOL software. The optimal mode with the lowest binding energy (Fig. 3C) displays the hydrogen bond interactions between the residues [F61 (2.6 Å), T62 (2.2 Å), S129 (2.5 Å), W154 (2.0 Å, 2.7 Å), and V177 (2.9 Å)] of the PET hydrolase and BHET, which are shown in the docked pose of the PET hydrolase–BHET complex. The 2D interaction map was generated using Ligplot+, which depicted the hydrophobic contacts of BHET with F208 and M130 of the PET hydrolase in red semicircles (Fig. 3D). The outcomes of protein structure and molecular docking analysis reinforce the significant potential of the PET hydrolase enzyme in plastic degradation. Additionally, these analyses shed light on the specific structural characteristics that enable and enhance its enzymatic activity in this regard.

### Diversity in the Structural Stability of PET Hydrolase in the Absence and Presence of BHET

**Fig. 4:**
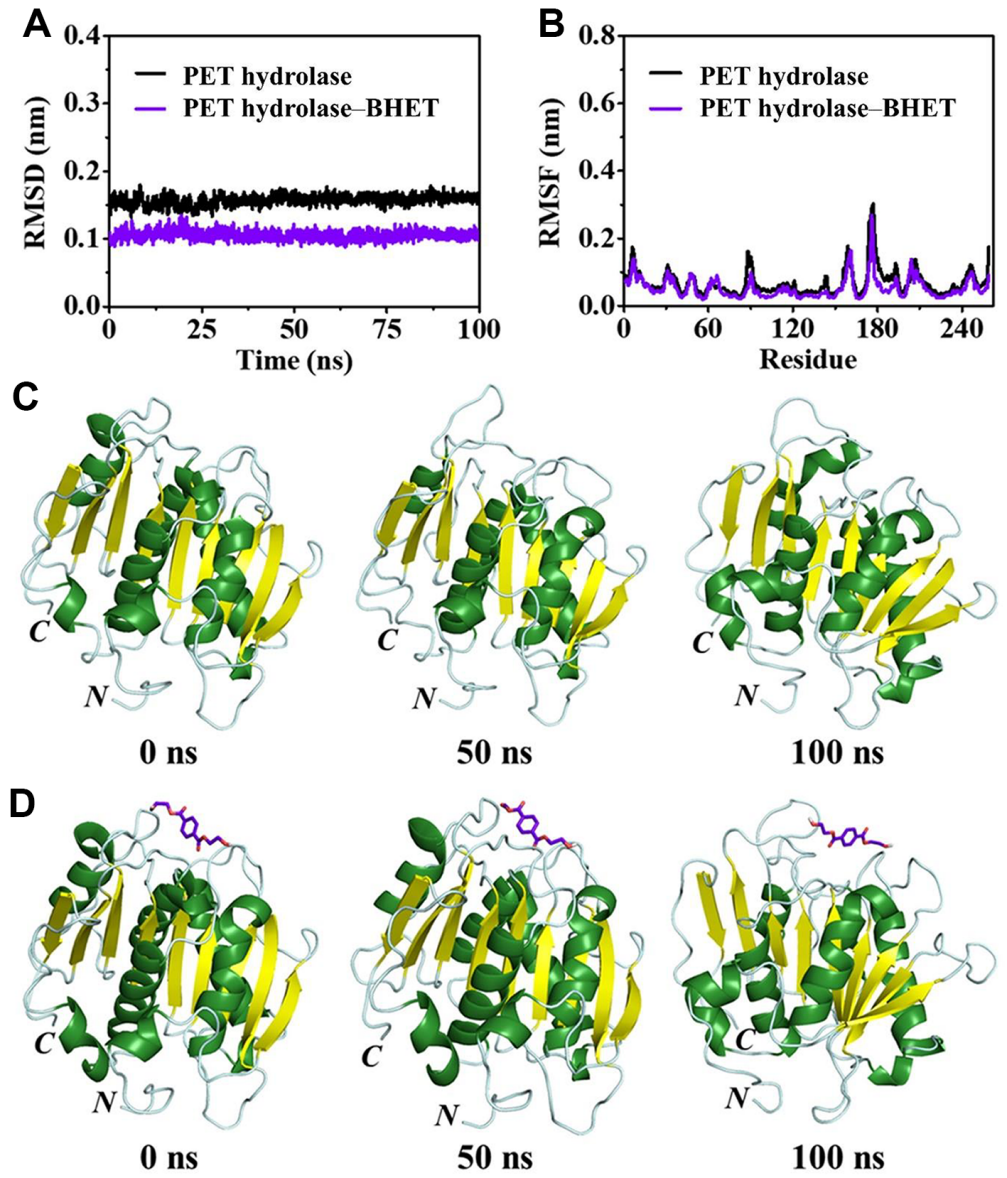
The RMSD and RMSF plots of PET hydrolase alone and PET hydrolase–BHET complex are shown in panels A and B. The conformational snapshots at various time points (0, 50, and 100 ns) for PET hydrolase alone and PET hydrolase–BHET complex are shown underneath (panels C and D, respectively).

The relative structural stability of the PET hydrolase alone and the PET hydrolase–BHET complex was illuminated using the structural parameters RMSD and RMSF. The average RMSD for the PET hydrolase alone was noted to be 0.157 ± 0.002 nm, while the average RMSD of the PET hydrolase–BHET complex oscillates at a significantly lower value (0.105 ± 0.001 nm) during simulation (Fig. 4A). The lower average RMSD of the PET hydrolase–BHET complex indicated the higher structure stability of the PET hydrolase in the presence of BHET as compared to the PET hydrolase alone.

The average RMSF for the PET hydrolase alone and the PET hydrolase–BHET complex was noted to be 0.069 ± 0.003 nm and 0.053 ± 0.002 nm, respectively (Fig. 4B). The RMSF analysis displayed lower fluctuations in ~93%residues of the PET hydrolase on the incorporation of BHET. The RMSF analysis illuminated reduced fluctuations in the residues N1–N11, E15–T47, G50–P59, F61, I68–L151, G153–T159, E162–L200, A205–T259 of the PET hydrolase on the inclusion of BHET. Notably, the reduced fluctuation in the residues of the PET hydrolase on the incorporation of BHET resulted in the lower flexibility and higher structural stability of the PET hydrolase.

In addition, we conducted a visual inspection of the conformational snapshots for the PET hydrolase alone and the PET hydrolase–BHET complex (Fig. 4C and 4D). The conformational snapshots extracted at various time points (0, 50, and 100 ns) from MD trajectories highlighted that BHET remained strongly bound to the binding site of the PET hydrolase during the whole simulation, and its binding was also predicted in the molecular docking studies (Fig. 4D).

### Impact of BHET on the Conformational Analyses of PET Hydrolase

The conformational clustering was conducted to anticipate the thermodynamic stability of the PET hydrolase alone and the PET hydrolase–BHET complex. In PET hydrolase alone, the percentage populations of the three most populated clusters (Cluster–1, Cluster–2, Cluster–3) were observed at 17.95, 9.75, and 8.2%, respectively (Fig. S2A). However, the percentage increased to 34.25, 10.85, and 9.25% for Cluster–1, Cluster–2, and Cluster–3, respectively, for the PET hydrolase on incorporating BHET (Fig. S2B). The top three clusters comprised 35.9% of the entire conformational ensemble in the PET hydrolase alone, whereas 64.35% of conformations were sampled in the three most-populated clusters of the PET hydrolase–BHET complex. Thus, the inclusion of BHET results in enhanced conformational uniformity within the PET hydrolase structure. The conformational clustering outcomes suggest the heightened thermodynamic stability in the PET hydrolase-BHET complex compared to PET hydrolase alone.

### Binding Free Energy Calculation between PET Hydrolase and BHET

**Fig. 5:**
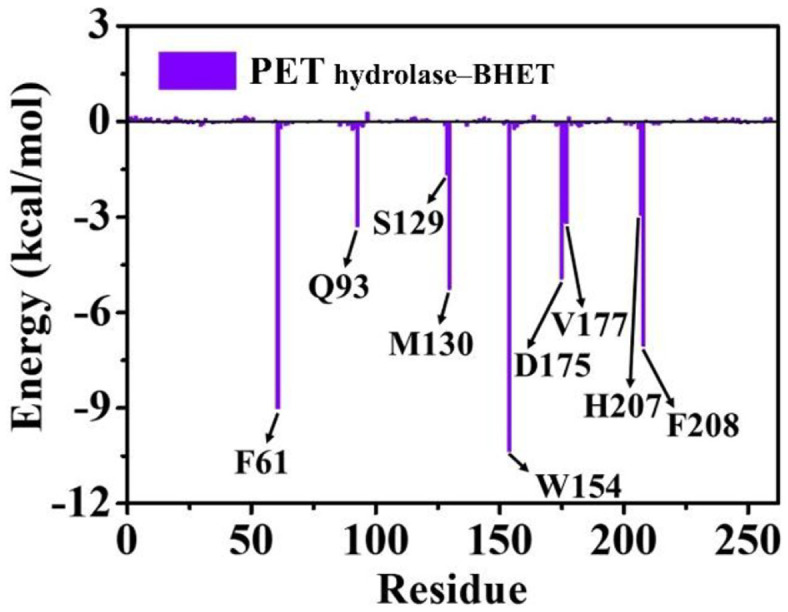
The residue-wise decomposed binding free energy of PET hydrolase–BHET complex.

BHET exhibited a robust binding to the PET hydrolase with a binding free energy of –37.4 ± 3.4 kcal/mol (Table 1). Notably, the van der Waals (Δ*E*_vdW_) interaction energy became a governing force in the binding of BHET to the PET hydrolase (–21.3 ± 1.7 kcal/mol). The electrostatic energy, Δ*E*_ele_ (–6.6 ± 1.3 kcal/mol) and non-polar solvation energy, Δ*G*_nps_ (–25.5 ± 4.3 kcal/mol), favorably contributed to the total binding free energy, whereas the polar solvation energy, Δ*G*_ps_ (16.0 ± 3.9 kcal/mol), was unfavorable in the binding of BHET with the PET hydrolase. The residue-wise binding free-energy decomposition analysis highlighted that F61 (–9.001 kcal/mol), Q93 (–3.276 kcal/mol), S129 (–1.646 kcal/mol), M130 (–5.252 kcal/mol), W154 (–10.343 kcal/mol), D175 (–4.924 kcal/mol), V177 (–3.188 kcal/mol), H207 (–2.924 kcal/mol), and F208 (–7.033 kcal/mol) of the PET hydrolase displayed strong binding with BHET (Fig. 5), which confirmed that the PET hydrolase residues play a major role in the binding of BHET with the enzyme.

### Protein Expression and Characterization of the Recombinant PET Hydrolase

The gene encoding the PET hydrolase was cloned into the pET-32a (+) expression vector and expressed in *E. coli* BL21(DE). His-tag was added to the N-terminus of the recombinant enzyme to facilitate purification. The soluble protein was purified by affinity chromatography. Three different concentrations of imidazole (10, 100, and 200 mM) were used to elute the protein. The soluble fractions are shown in (Fig. S3). The recombinant PET hydrolase had an estimated molecular mass of approximately 51.31 kDa by SDS-PAGE, which corresponds to the value obtained by calculating the amino acid sequence of the enzyme.

**Fig. 6:**
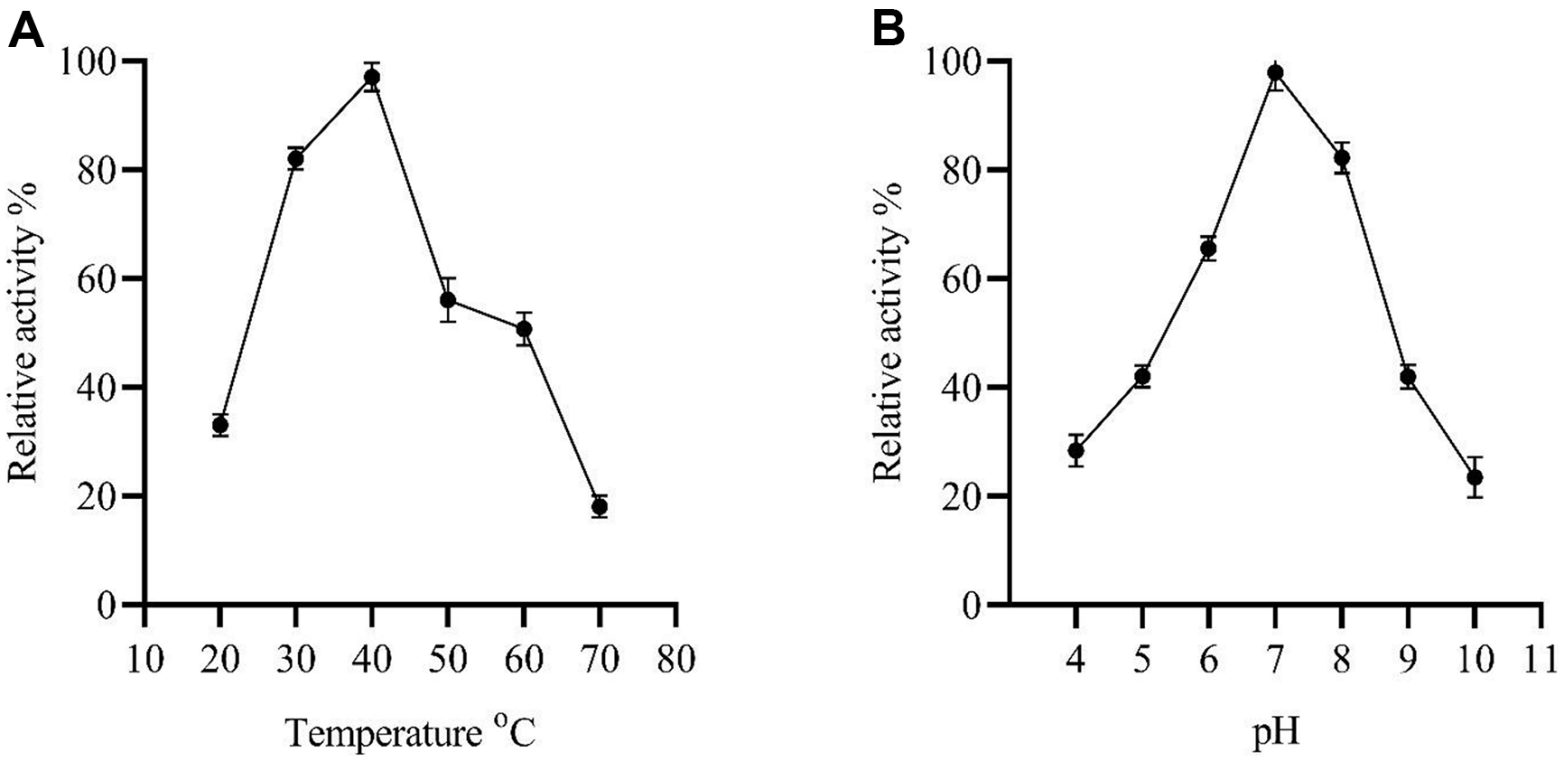
The effect of temperature and pH on PET hydrolase activity and stability. The relative activity of purified PET hydrolase was determined at different pH values and temperature by using p-NP butyrate (p-NPC_4_) as the substrate at 405 nm.

Enzymatic properties can be influenced by temperature and pH values. The effect of temperature on the PET hydrolase activity was investigated from 20°C to 70°C at a pH of 7.0 (Fig. 6A). The maximum activity was observed at 40°C. The relative enzyme activity after 120 min of incubation at 30°C, 50°C, and 60°C was approximately 82, 56 and 60%, respectively, indicating that the PET hydrolase was stable between 30°C and 60°C. However, the enzyme was unstable above 60°C and retained only about 18% of its activity, probably due to thermal denaturation.

In addition, the effect of pH on the PET hydrolase was tested in a pH range of (4.0 to 10.0) at 40°C (Fig. 6B). The maximum activity was observed at a pH of 7.0. The enzyme’s activity was highly sensitive to pH changes around the optimal value (pH 7.0). At pH below 5.0 and above 9.0, its activity did not exceed 40%. Optimal enzyme activity was achieved at a pH of 7.0, while it was almost inactivated at a pH of 4.0. After a 120 min incubation at a pH of 7.0–8.0, the relative activity of the enzyme remained at about 70% compared to the initial activity. At pH 4.0 and 10.0, only 28% and 23% of the activity was retained. Thus, the PET hydrolase was relatively stable in neutral and slightly alkaline environments.

Metal ions and organic solvents often serve as potential inhibitors or activators of enzymes. As shown in (Table 2), positive monovalent alkali metals and divalent alkaline earth metals had a lower inhibitory effect on the PET hydrolase than transition metals. Zn^2+^ and Cu^2+^ showed a significant inhibitory effect on the enzyme, with 25± 0.43% and 18.51 ± 1.76% relative activity, respectively. Fe^3+^, K^+^, and Ba^2+^ had no obvious effect on enzyme activity, while the positive bivalent alkaline earth metal Mg^2+^ activated the PET hydrolase with a relative activity of 110.7 ± 0.06%. In addition, organic compounds such as Tween 20 (1%) strongly inhibited the PET hydrolase with a relative activity of 71.2 ± 0.15%. Acetonitrile (1%), EDTA (1%) and dimethyl sulfoxide (1%) showed moderate activation of enzyme activity, while methanol (1%) and ethyl acetate (1%) had little effect on the PET hydrolase with a relative activity of 125.9 ± 0.23% and 114.8 ± 0.82%, respectively.

### Identification of Product from BHET Degradation

**Fig. 7:**
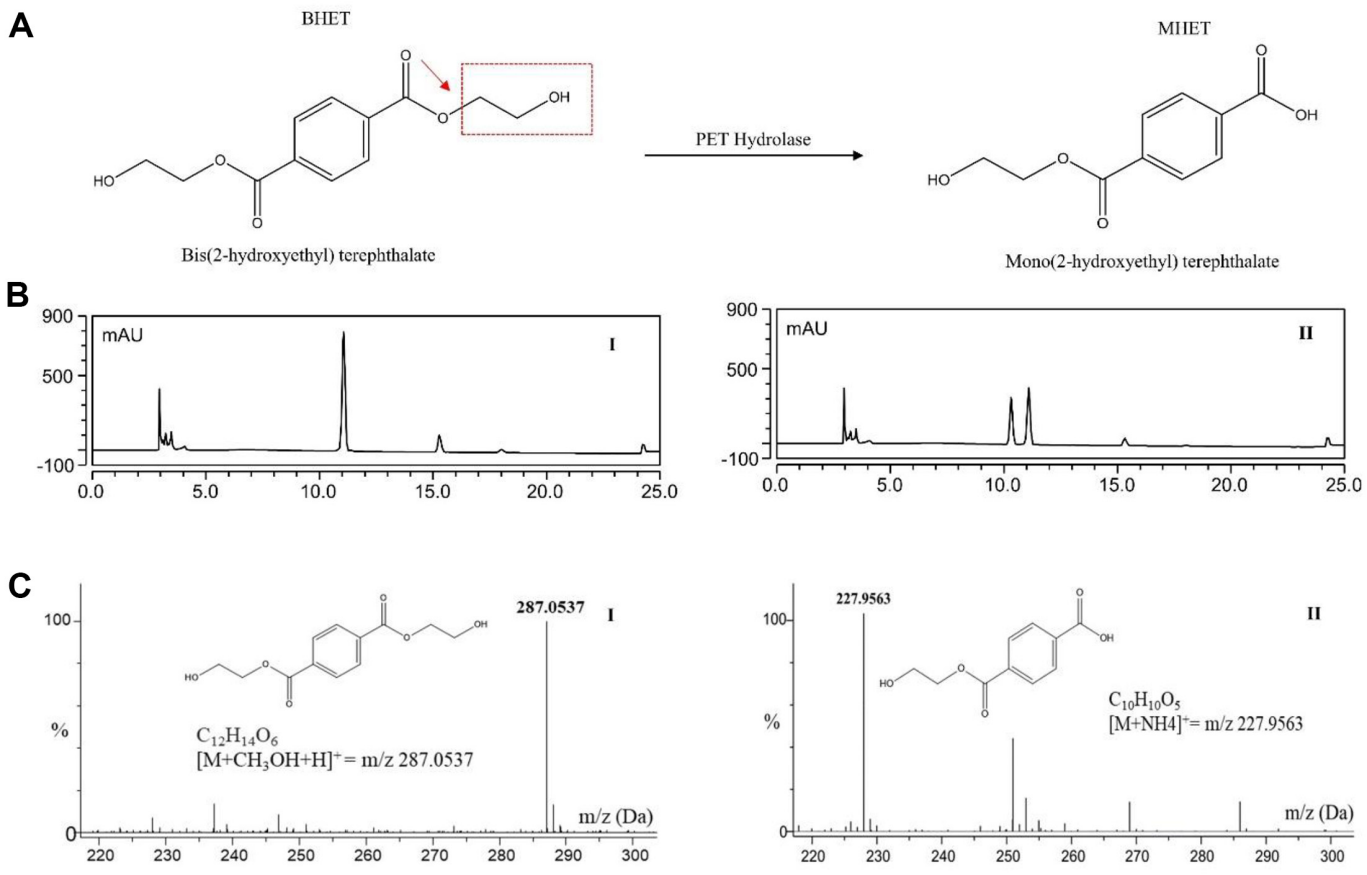
(A) The degradation of BHET by PET hydrolase. Acyl-oxygen bond is shown by red arrows and hydrolyzed glycol group is shown by red box. (**B**) I and II, HPLC chromatogram of standard BHET and degraded product MHET, respectively. (**C**) I and II, HR-QTOF ESI/MS analysis of degradation product MHET from BHET.

PET hydrolases, constituting carboxylic ester hydrolases, demonstrate the ability to hydrolyze BHET due to their water solubility. Despite a low sequence identity, these enzymes have a remarkably similar function [60]. The mechanism of BHET hydrolysis begins when PET hydrolases consume the plastic polymer and degrade it into more readily adaptable monomers. Under these conditions, the microorganisms can take up the plastic monomers as their main carbon sources, which are then converted into CO_2_, H_2_O, CH_4_, and N_2_ [61]. To identify the degradation product of BHET degradation by the PET hydrolase, the degraded product was detected by high-performance liquid chromatography and the product was confirmed by mass detection. In HPLC chromatograms, the standard, BHET, and the product MHET were detected at retention times of 11 min and 10.5 min, respectively (Fig. 7B). Meanwhile, HR-QTOF ESI/MS analysis showed the degradation product MHET from BHET (Fig. 7C). The profile of the depolymerization products of the PET hydrolase was similar to that of other bacterial PET hydrolases and cutinases. The main product is MHET, and while IsPETase in *I. skaieneis* produces mainly MHET, a second extracellular enzyme, MHETase, is required for the conversion of MEHT to TPA. Subsequently, a specialized transporter in the periplasm imports the TPA into the cytoplasm [14]. The degree of depolymerization by the PET hydrolase was 38% after 30 min. The conversion rate of BHET by Trx-IsPETase was reported to be 39.34% [62]. Although the difference in conversion rate was not remarkable, the percentage of product formation by PET hydrolase was almost similar to that of Trx-IsPETase. The limited accessibility and hydrophobic nature of crystalline PET, as well as the sensitivity of the enzyme to temperature, pH, and specificity, create challenges for existing PET hydrolases. These variables prevent PET from being effectively degraded, even when it is widely distributed in the environment.

## Conclusion

In this work, we report for the first time the biochemical characterization and heterologous expression of *Streptomyces* sp. W2061 PET hydrolase in *E. coli*. The PET hydrolase successfully degraded a single ester bond of BHET, resulting in the formation of MHET, which was analyzed by LC-MS. Additionally, docking and MD simulations were performed, enabling us to obtain a molecular-level interpretation of the substrate in the active site. These simulations explored conformational changes and interaction dynamics over time, emphasizing the potential of our PET hydrolase to degrade plastics and highlighting the structural features that may facilitate enzymatic activity. Several PET hydrolases have already been discovered, and there is a current need to modify them to enhance their ability to break down BHET’s ester linkage. Therefore, this research focused on a detailed study of PET hydrolase with BHET, both *in vitro* and in silico, thus emphasizing BHET-degrading microbial hydrolases and their role in reducing microplastic pollution in the environment.

## Supplemental Materials

Supplementary data for this paper are available on-line only at http://jmb.or.kr.



## Figures and Tables

**Table 1:** The binding free energy components calculated using g_mmpbsa with MM–PBSA method.

Energetic components	Binding free energy (kcal/mol)
Δ*E*_vdw_	– 21.3 ± 1.7
Δ*E*_elec_	–6.6 ± 1.3
Δ*E*_MM_^a^	–27.9 ± 3.0
Δ*G*_ps_	16.0 ± 3.9
Δ*G*_nps_	–25.5 ± 4.3
Δ*G*_solv_^b^	–9.5 ± 0.4
Δ*G*_binding_^c^	–37.4 ± 3.4

^a^Δ*E*_MM_ = Δ*E*_vdw_ + Δ*E*_elec_; ^b^Δ*G*_solv_ = Δ*G*_ps_ + Δ*G*_nps_; ^c^Δ*G*_binding_ = Δ*E*_MM_ + Δ*G*_solv_

**Table 2:** The effect of various metal ions and organic solvents on enzyme activity.

Metals and other compounds	Relative activity (%)
Control	100
Positive monovalent light alkali metals	
NaCl	82.1 ± 2.4
KCl	94.3 ± 2.3
NH_4_Cl	80.2 ± 2.9
Positive bivalent alkali earth metal	
MgCl_2_	110.7 ± 0.06
BaCl_2_	99.3 ± 0.19
Transition metals	
ZnCl_2_	25 ± 0.43
FeCl_2_	103 ± 0.12
CuSo_4_	18.51 ± 1.76
Organic solvents	
Methanol	125.9 ± 0.23
Acetonitrile	97.8 ± 1.75
Ethyl acetate	114.8 ± 0.82
Tween 20	71.2 ± 0.153
DMSO	107.4 ± 0.32
EDTA	85.3 ± 2.6

The reaction system included 120 ul ρ-NPC4 ester substrates (10 mmol/l), 1.68 ml Tris-HCl buffer (50 mmol/l, pH 7.0), and 200 ul purified PET hydrolase solution, containing 1 mM of different metals and 1.0% volume concentration of an organic solvent. The control activity was determined without the addition of metal ions or organic solvents.
