# Supplementary material for: In Silico Analysis and Biochemical Characterization of Streptomyces PET Hydrolase with Bis(2-Hydroxyethyl) Terephthalate Biodegradation Activity
Source: J Microbiol Biotechnol. 2024 Jul 25;34(9):1836–47. doi: 10.4014/jmb.2404.04030 (PMC11485624; doi:10.4014/jmb.2404.04030)
Supplement: Supplementary file 1 [file jmb-34-9-1836-supple.pdf]

J. Microbiol. Biotechnol. 2024. 34(9): 1836–1847

https://doi.org/10.4014/jmb.2404.04030

Supplementary Table and Figures

In-silico analysis and biochemical characterization of *Streptomyces* PET hydrolase with bis(2-hydroxyethyl) terephthalate biodegradation activity

Gobinda Thapa[1], So-Ra Han[2], Prakash Paudel[1], Min-Su Kim[1], Young-Soo Hong[3], and Tae-Jin Oh[1,2,4,5,*]

[1]Department of Life Science and Biochemical Engineering, SunMoon University, Asan 31460, Republic of Korea; gv.thapa2009@gmail.com (G.T.); prakukcde2016@gmail.com (P.P.); galun12@naver.com (M.-S.K.)

[2]Bio Big Data-Based Chungnam Smart Clean Research Leader Training Program, SunMoon University, Asan 31460, Republic of Korea; 553sora@hanmail.net (S.-R.H.)

[3]Chemical Biology Research Center, Korea Research Institute of Bioscience and Biotechnology, Ochang 28116, Republic of Korea; hongsoo@kribb.re.kr (Y.-S.H.)

[4]Genome-Based BioIT Convergence Institute, Asan 31460, Republic of Korea

[5]Department of Pharmaceutical Engineering and Biotechnology, SunMoon University, Asan 31460, Republic of Korea

[*]Author for correspondence: Prof. Tae-Jin Oh

E-mail: tjoh3782@sunmoon.ac.kr

***Streptomyces* sp. PET hydrolase nucleotide sequence**

GTGCAGCAGCACCTCCCCTCCGGCACCATCCCCCCGCACCCTTCCCGCCCGAGCCGCTCCGG
TACGTTCACGGGTCGCTCCCGTACGCTGACGGGTCTGGTGACCGCCGGTGCGGCCACCGCCG
GCCTCCTGCTGACGGGCCTGGCGCCCGGCGCCCAGGCCGCCGACAATCCCTATGAGCGCGGC
CCGGCTCCCACCAACGCCTCCATCGAGGCGAGCCGCGGCTCGTACTCCACTTCCCAGACCTC
GGTGTCCTCGCTCGCCGTGACCGGATTCGGCGGCGGCACCATCTACTACCCGACGTCCACCG
CGGACGGCACCTTCGGCGCGGTCGTCATCTCGCCCGGCTTCACCGCTTACGAGTCCTCGATC
GCGTGGCTGGGACCACGCCTGGCCTCCCAGGGCTTCGTCGTGTTCACCATCGACACCAACAC
CACGCTCGACCAGCCCGACAGCCGGGGCCGCCAACTCCTCGCCGCCCTGGACTACCTGACCC
AGCGCAGTTCGGTACGGACCCGGGTCGACGCCGGACGCCTCGGTGTGATGGGCCACTCGATG
GGCGGCGGTGGCTCCCTGGAGGCCGCCAAGAGCCGCACATCGCTGAAGGCGGCGATCCCGCT
GACCGGCTGGAACACCGACAAGACCTGGCCCGAACTGCGCACGCCCACCCTCGTGGTGGGGG
CGGACGGTGACACCGTCGCCCCGGTCGCCACGCACTCCGAACCGTTCTACGAGTCGCTGCCC
GGCTCCCTCGACAAGGCGTACCTGGAGCTGCGCGGCGCCTCGCACTTCACGCCGAACACCTC
CGATACGACGATCGCCAAGTACAGCATCTCCTGGCTGAAGCGATTCATCGACAATGACACCC
GCTACGAGCAGTTCCTCTGCCCGCTCCCGCGGCCGAGCCTGACCATCGCGGAGTACCGGGGC
ACCTGCCCGCACACGGCGTAG

***Streptomyces* sp. W2061 PET hydrolase** (GenBank accession no WP 030719063.1)

MQQHLPSGTIPPHPSRPSRSGTFTGRSRTLTGLVTAGAATAGLLLTGLAPGAQAADNPYERG
PAPTNASIEASRGSYSTSQTSVSSLAVTGFGGGTIYYPTSTADGTFGAVVISPGFTAYESSI
AWLGPRLASQGFVVFTIDTNTTLDQPDSRGRQLLAALDYLTQRSSVRTRVDAGRLGVMGHSM
GGGGSLEAAKSRTSLKAAIPLTGWNTDKTWPELRTPTLVVGADGDTVAPVATHSEPFYESLP
GSLDKAYLELRGASHFTPNTSDTTIAKYSISWLKRFIDNDTRYEQFLCPLPRPSLTIAEYRG
TCPHTA

Fig. 1S. *Streptomyces* sp. W2061 PET hydrolase gene encoding nucleotide sequence and corresponding amino acid sequence of PET hydrolase.

Table S1. Sequence similarity and identity to others PET hydrolases.

Enzyme		Identity				
		PET hydrolase W2061	Lipase (1JFR)	Cutinase Est119 (3VIS)	PET hydrolase (4CG1)	Alpha beta hydrolase (7YKQ)
PET hydrolase W2061	**Similarity (%)**	100	78.8	55.1	48.4	56.3
Lipase (1JFR)		81.3	100	53.2	57.9	68.5
Cutinase Est119 (3VIS)		64.7	62.9	100	65.7	55.8
PET hydrolase (4CG1)		58.4	70.5	71.6	100	56.7
Alpha beta hydrolase (7YKQ)		66.2	79.3	67	70.6	100

Sequence identity and similarity percentage of PET hydrolase W2061 from the lipase (1JFR, *Streptomyces exfoliates*), Cutinase Est119 (3VIS, *Thermobifida alba*), PET hydrolase (4CG1, *Thermobifida fusca*), and Alpha beta hydrolase (7YKQ, *Thermomonospora curvata* DSM43183) were calculated from pairwise alignments of proteins using Emboss Needle software.

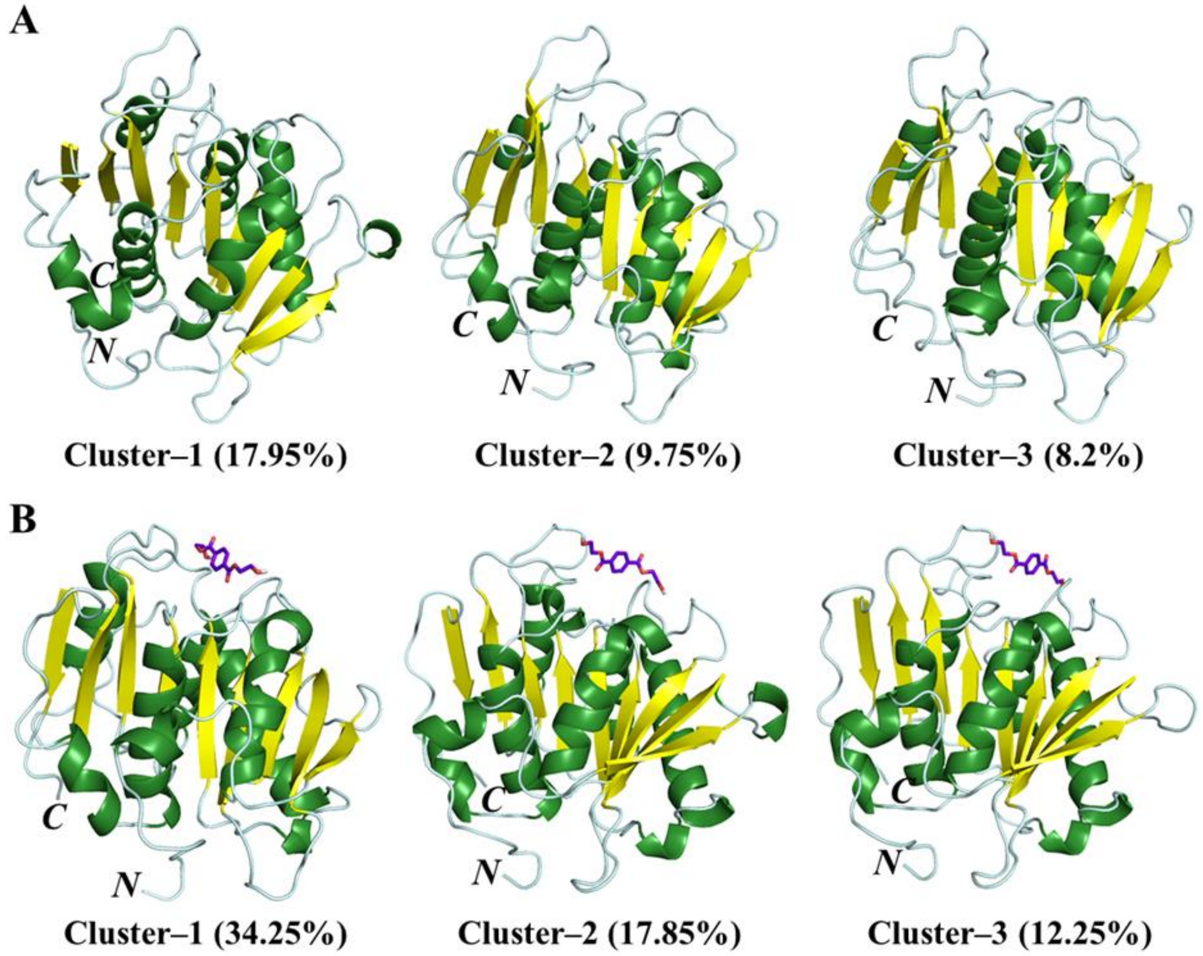

Fig. S2. The representative members of the most-populated conformational clusters for PET hydrolase alone and in the presence of BHET are displayed in panel a, and b with their percentage population.

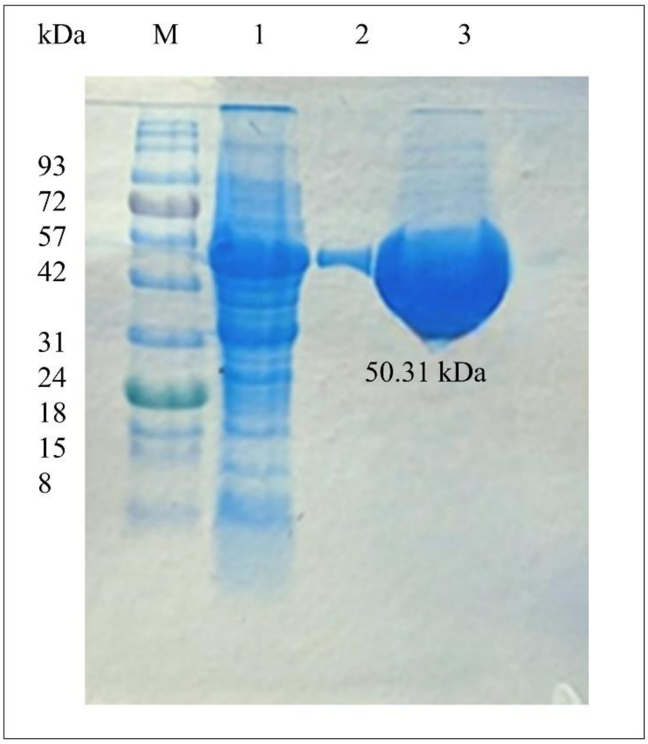

Fig. 3S. The 15% SDS-PAGE analysis for the recombinant PET hydrolase, expressed in *Escherichia coli* BL21 cells after purification. M, molecular weight marker; 1, lysate supernatant; 2, 200 mM elution fraction; and 3, purified PET hydrolase.
